# Case Report: Pathological Personality Traits Through the Lens of the ICD-11 Trait Qualifiers and the DSM-5 Section III Trait Model: Two Patients Illustrating the Clinical Utility of a Combined View

**DOI:** 10.3389/fpsyt.2021.627119

**Published:** 2021-03-09

**Authors:** Tim Bastiaens, Dirk Smits, Laurence Claes

**Affiliations:** ^1^Department of Diagnostics, University Psychiatric Center KU Leuven, Kortenberg, Belgium; ^2^Faculty of Psychology and Educational Sciences, KU Leuven, Leuven, Belgium; ^3^Department of Research and Project Management, Odisee University College, Brussels, Belgium; ^4^Faculty of Medicine and Health Sciences, University of Antwerp, Antwerp, Belgium

**Keywords:** personality disorders, personality traits, ICD-11, DSM-5, PID5BF+ M

## Abstract

We report on two individuals presenting for treatment as part of everyday clinical practice, comparing their pathological personality traits through the lens of the ICD-11 trait qualifiers and the DSM-5 Section III personality trait model. We compare higher order pathological personality domains and lower order pathological personality trait facets of patient M (diagnosed with borderline personality traits according to DSM-5 Section II), and patient L (diagnosed with obsessive-compulsive personality traits according to DSM-5 Section II) with normative data and with each other. Findings highlight the clinical utility of a ICD-11/DSM-5 combined view, including: (1) the Disinhibition/Anankastia personality domain distinction as advocated in the ICD-11 model, (2) the Psychoticism personality domain as conceptualized in the DSM-5 Section III personality trait model, as well as (3) the use of lower order personality trait facets within each higher order personality domain.

## Introduction

Both the 11th edition of the International Classification of Diseases [ICD-11; (1)] and the fifth edition of the Diagnostic and Statistical Manual [DSM-5; (2)] contain personality pathology models. In DSM-5, two models are represented: a traditional categorical approach in section II, and a dimensional Alternative Model for Personality Disorders (AMPD) in section III. The latter provides a criterion A expressing severity through the Levels of Personality Functioning Scale [LPFS (3), ranging from no impairment (0), over mild (1), moderate (2), severe (3), to extreme (4) impairment], and a criterion B expressing descriptive pathological personality traits. Criterion B is separately referred to as the DSM-5 Trait model and is operationalized through the Personality Inventory for DSM-5 [PID-5; (4)]. In parallel to the AMPD, the ICD-11 also distinguishes between Personality Disorder Severity (ranging from None, over Personality Difficulties, Mild Personality Disorder, Moderate Personality disorder, to Severe Personality Disorder), and descriptive personality trait domains, labeled Qualifiers. In addition, the ICD-11 also contains an optional “borderline pattern qualifier,” following the DSM-5 categorical description of borderline personality disorder.

The trait domains defined by both the ICD-11 and the DSM-5 are to a great extent commensurate, as both contain highly similar Negative Affectivity, Detachment, and Disinhibition dimensions. The ICD-11 Dissociality domain further parallels the DSM-5 Antagonism domain, albeit with a stronger focus on dissocial behavior and traits in the former (5). Contrary to the DSM-5 Trait model however, the ICD-11 contains an additional Anankastia domain, representing a Compulsivity dimension that, in the final DSM-5 Section III personality trait model, was subsumed under the Disinhibition domain (4). Contrary to the ICD-11 model, the DSM-5 Section III personality trait model comprises a Psychoticism domain, which in ICD-11 terms is considered part of the syndromal schizophrenia spectrum and thus not conceptualized as a separate personality dimension. Finally, the ICD-11 model does not provide further specifications through the use of personality trait facets, as the DSM-5 Section III model does.

Recent studies have documented the convergent validity of both models (5), but also point to potential shortcomings (6). First, the Disinhibition domain of the DSM-5 Trait model has been known to align more with Antagonism than with Compulsivity (7). Second, contrary to the current ICD-11 model, recent findings strongly advocate the conceptualization of a separate psychoticism personality domain (8). Third, lower-order trait facets, not present in the current ICD-11 model, have been shown to explain variance additional to their higher-order domain (9).

A synchronization of both models could benefit clinical utility (10), in providing more complete diagnostic coverage (11) and allowing for a more person-tailored case conceptualization and subsequent treatment approach. Recently, a measure combining both conceptualizations was developed starting from the item pool of the PID-5 and using ant colony optimization algorithms (12). The resulting Personality Inventory for DSM-5, Brief Form Plus (PID5BF+) was subsequently adapted to also capture trait facets of the ICD-11 Anankastia domain, leading to the Modified Personality Inventory for DSM-5—Brief Form Plus [PID5BF+ M; (6)].

In the current report, we compare the PID5BF+ M higher and lower order personality traits of two treatment-seeking individuals L and M with normative data and to each other. Our aims are to investigate: (1) whether maladaptive trait expressions are useful sources of information for case formulation and treatment planning, (2) whether the Disinhibition/Anankastia personality domain distinction as advocated in the ICD-11 model has clinical utility, (3) whether the Psychoticism trait domain as conceptualized in DSM-5 has clinical utility, and (4) whether the use of lower order personality trait facets has clinical value beyond trait domains.

## Case Presentation

**Figure 1:**
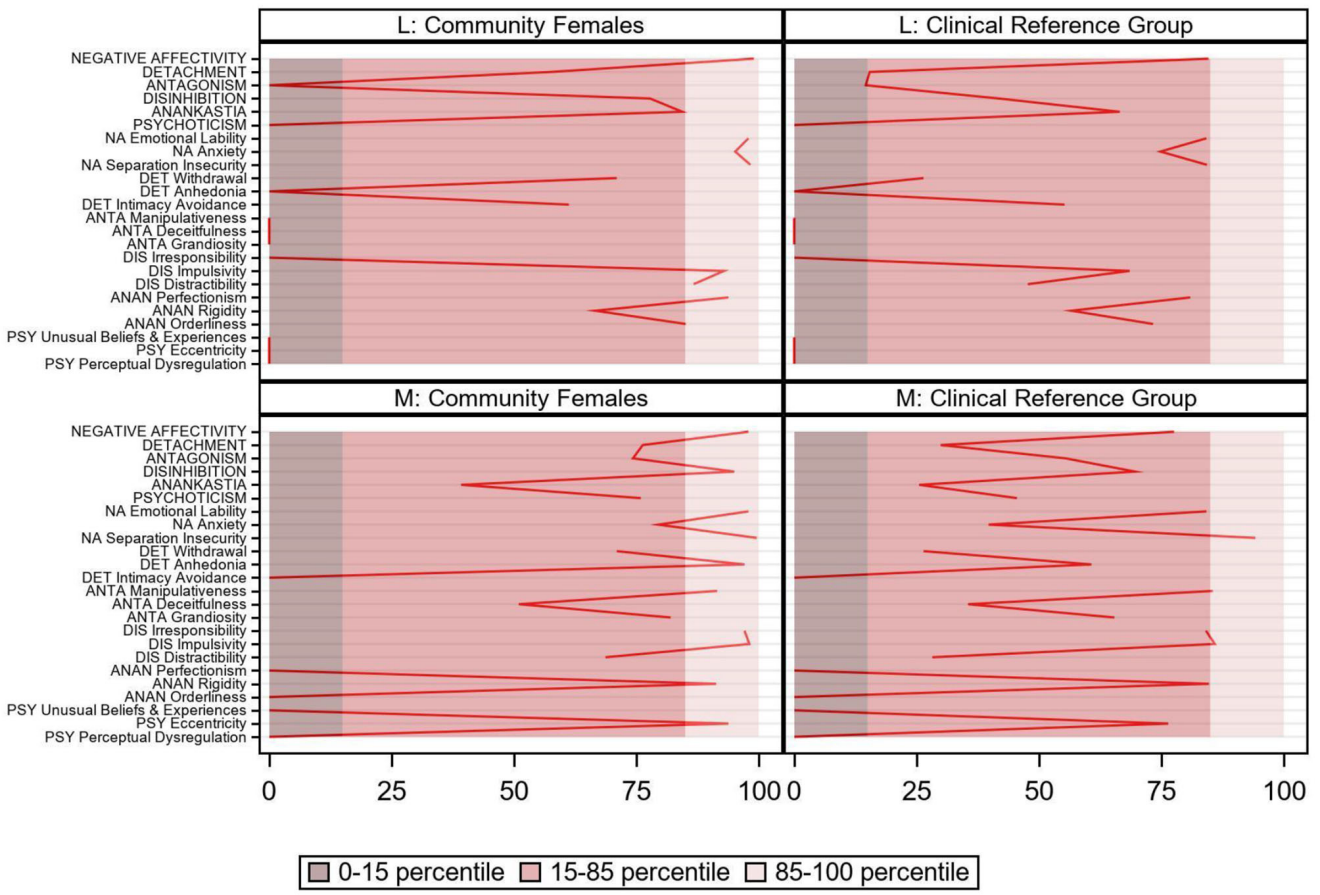
PID5BF+ M pathological personality trait domains and facets for patients L and M, community [*N* = 482; (13)] and clinical [*N* = 244; (7)] reference group.

**Figure 2:**
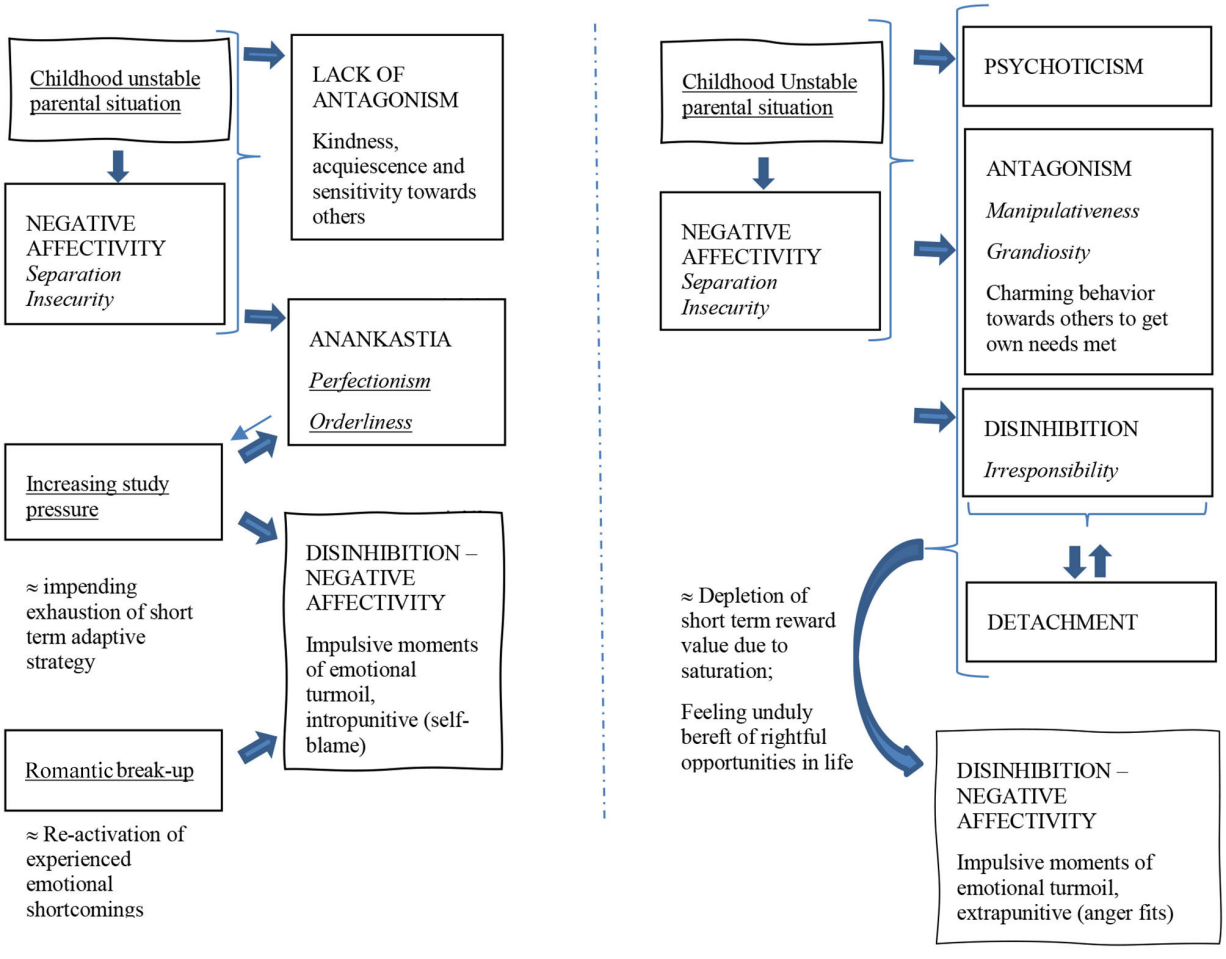
Two possible hypothetical case formulations (left: L, right: M), based on PID5BF+ M domain (capitals) and trait scores (italic) and life history narratives (underscored); thick arrows indicate a further promotion/triggering of given personality traits.

Table 1 summarizes background features of L and M. Both were admitted to an Inpatient Psychiatric Hospital in Belgium, in order to obtain a multidisciplinary assessment of experienced intra- and interpersonal problems. Neither presented with acute psychiatric symptoms at the time of hospital admission. The individual as well as family history of both patients revealed no relevant somatic or mental illnesses or genetic predispositions. M had briefly consulted a psychiatrist four months before current hospitalization on account of experienced moodiness. Three weeks before hospital admission, L had attended two group sessions with the faculty counselor as part of a performance anxiety course she subsequently dropped. Both patients were informed of and agreed to the anonymous use of their assessment data in the current case report through a signed informed consent, in accordance with the CARE guidelines. To further ensure anonymity, life history specifics were substituted by factitious parallel information. As part of hospital policy, the results were discussed at length with each patient upon completion of the assessment, including a presentation of the hypothetical case formulation derived from Figure 1 and presented in Figure 2. Both patients experienced the received feedback as illuminating and helpful, as documented in their electronic patient file. The current case report was approved by the Ethical Committee of the hospital.

**Table 1:** Descriptive background features of patients L and M.

	**L**	**M**
Gender	Female	Female
Age	19 years	20 years
Level of education	High school degree	High school degree
Family and living situation	One younger sister Living with mother and stepdad	Four sisters and two brothers Living with mother Father deceased at K's early age
Current occupation	1st year graduate student in biomedical sciences	Part time job in a second-hand record store

*Patient L* presented with intermittent outbursts of emotional dysregulated behavior in daily life, manifesting through re-occurring anxiety and suicidal ideation. These outbursts were experienced as impulsive moments of acute emotional turmoil, with an intensity L herself could not fully account for. During the initial conversation, L struck the interviewer as rather docile and ingratiating. The clinical interview further revealed that, according to L, mother and stepdad were very close and supportive but also suffering because of L's problems. Mother in particular was experienced by L as easily overwhelmed and quick to indirectly complain, adhering to a victim-like presentation for which L subsequently felt even more responsible. L further experienced difficulties with her biological father, who left mother at L's birth and who sought reconciliation at patient's eight years of age. Although L felt that he was not very mature and still didn't act much as a responsible parent, she agreed to his persistence on seeing her regularly, because this enabled her to spend time with her paternal grandmother, who, although of old age and thus to some degree frail as well, was L's secure attachment figure. L looked for proximity in peers, by whom she was considered a very friendly, acquiescent but cheerful, and upright person, sensitive to the needs and griefs of others. As a methodical individual, she studied hard as ever, but increasingly experienced difficulties meeting higher expectations. L developed an eating disorder for a while. She quit volleyball practice on account of her idea on ruining things for her teammates because of her bad performances—to which her team disagreed. In her postgraduate biomedical studies, L experienced trouble concentrating. She was failing several classes and losing motivation, which contrasted with her orderly and perfectionistic demeanor during high school studies. L had been head-over-heels involved in one romantic relationship, that ended abruptly and left L oscillating between feelings of despair and clinging behavior.

*Patient M* presented with re-occurring moments of emotional dysregulated behavior in daily life as well, in her case manifesting through anger fits. In the clinical interview, M further stated that she experienced her family situation as rather turbulent and quarrelsome, and resented that her mother did not grant her the financial resources to start a higher education. Her spare time was largely spent as an active member in a youth hangout bar, amongst friends. M stated to have multiple romantic relationships and to be flirtatious with boys out of proclaimed proximity needs. She described rather acquiescent behavior toward female peers, but also admitted to deliberately charming behavior toward boys, to get them to meet her wishes. Her re-occurring moments of emotional dysregulated behavior through anger fits occurred when things didn't go her way and frustration overwhelmed her, mostly occurring in the presence of mother or boyfriends. From the interviewer's first impression, M came across as somewhat whimsical and unconventional, displaying an apathetic laissez-faire attitude and presenting society as unduly denying career opportunities to people like herself. In contrast, she did value her friendships strongly, and was able to sustain part time employment at a second hand record store, where she was considered a bit of a weird one, but was nonetheless quite liked by her two colleagues and boss, despite her frequent tardiness. M described cannabis and alcohol use with peers as escaping as well as hedonistic behavior.

The Semi-structured Interview for DSM-5 Personality Functioning [STiP-5.1; (14)] situated both L and M at a moderate level of impairment in personality functioning according to the DSM-5 Section III criterion A. In terms of ICD-11 Personality Disorder Severity, L and M classified as having a Mild Personality Disorder. On the Structured Interview for DSM-5 Section II Personality Disorders [SCID-5-P; (15)], L met four out of the eight criteria for the DSM-5 Section II obsessive-compulsive personality disorder, thereby scoring below the threshold. M met three out of nine criteria for the DSM-5 Section II borderline personality disorder, thereby scoring below threshold for the categorical diagnosis as well. Combined with their life history narratives, we expected L to score high on the PID5BF+ M personality trait domains Negative Affectivity and Anankastia, and M to score high on Negative Affectivity and Disinhibition.

## Diagnostic Assessment

### Modified Personality Inventory for DSM-5—Brief Form Plus [PID5BF+ M; (6)]

The PID5BF+ M is a self-report questionnaire that represents a shortened and modified version of the original PID-5. It consists of 36 items and provides six higher order pathological personality trait domains, namely Negative Affectivity, Detachment, Antagonism, Disinhibition, Psychoticism, and Anankastia. Each domain comprises three pathological personality trait facets, listed in Figure 1 (for example, Negative Affectivity comprises the trait facets Emotional Lability, Anxiety, and Separation Insecurity). Each personality trait facet encompasses two items, scored on a scale from 0 (*not at all true*) to 3 (*entirely true*). The construction process of the PID5BF+ M is described in Kerber et al. (12). Its validity has been documented in 15 countries (6).

## Results

Figure 1 shows PID5BF+ M trait domain and facet percentile scores in comparison to the Dutch community reference group. As hypothesized, both patients scored very high on Negative Affectivity (L: pc99 and M: pc97.9), L but not M showed an elevated (pc84.4) Anankastia score, and M (pc95) but not L scored high on Disinhibition. Surprisingly perhaps, L's Disinhibition score was moderately elevated as well (pc77.8). Consistent with their respective life history narrative, L displayed bottom scores on Antagonism and Psychoticism, in contrast to M's above average scores (resp. pc74.3 and pc75.9). On Detachment, M but not L exhibited a moderately elevated score (pc76.3). Regarding trait facets, some interesting clarifications emerged. For example, M's Detachment domain comprised high Anhedonia (pc97.1) and a bottom score on Intimacy Avoidance, contrasting L's trait facet profile. Both M's and L's Disinhibition domain consisted of high Impulsivity (pc98.1 and pc92.9), but this was supplemented with a high (L: pc97.1) vs. bottom (M) Irresponsibility score. Regarding the Anankastia domain, M showed bottom scores on Perfectionism and on Orderliness, while scoring high on Rigidity (pc91.3). In contrast, L exhibited high Perfectionism (pc93.8) and high Orderliness (pc85.1), while scoring average on Rigidity. Finally, M's above average score on the Psychoticism domain was solely due to a high score on Eccentricity (pc93.8). In comparison to the clinical reference group, M's and L's trait domain and facet scores displayed a very similar pattern, all situated somewhat lower (Figure 1).

## Discussion

The current clinical case report illustrates how combining the DSM-5 Trait model and the ICD-11 personality trait qualifiers perspective can lead to a better understanding of patients individually, as well as of the similarities and differences between them. Firstly, distinguishing between a Disinhibition and an Anankastia personality domain as advocated in the ICD-11 model, allowed L's Anankastia to come into focus, that in a DSM-5 Trait model view would have been subsumed under the Disinhibition dimension and, as both ICD-11 domains contribute inversely to DSM-5 Section III Disinhibition, would have occluded both L's level of Anankastia as well as her level of Disinhibition. Secondly, the addition of the DSM-5 Psychoticism domain to the ICD-11 model allowed M's Eccentricity, in this case clearly to be conceptualized as a personality trait rather than a part of a syndromal schizophrenia spectrum, to show itself. Thirdly, the unfolding of each higher order personality domain in comprising personality trait facets allowed to conceptualize important differences between both L's and M's elevated Disinhibition domains, with clinically important differences in Irresponsibility. Also, it allowed clarification of M's normatively below average instead of very low Anankastia, as the from the narrative expectedly very low Perfectionism and expectedly very low Orderliness was tempered by high Rigidity, in this case associated with persistence and frustration regarding having things her way.

Integrating PID5 BF+ M results with life history narratives further enables us to formulate hypothetical working models depicting possible functional interactions [(16); Figure 2], that can be the starting point of individually tailored therapeutic interventions (17, 18). For L, we could for example think of her unstable parental situation during childhood, leading to heightened separation insecurity, as a context in which she further resorted to acquiescence and strong investment in others in order not to make matters worse. At the same time becoming even more sensitive to internal standards, both morally and in terms of performance, L developed a perfectionism and an inclination toward orderliness leading to an increase of academic pressure. With the romantic break-up triggering a re-activation of experienced emotional shortcomings, L experiences re-occurring outbursts of anxiety and suicidal ideation. From this hypothetical case formulation, targeting L's acute moments of emotional turmoil will imply addressing her inclination toward compulsivity as well her at first glance very adaptive agreeable behavior, as her inclination to deal with unmet emotional needs.

For M, disadvantageous childhood experiences may have led to heightened separation insecurity as well, but in contrast to L's case, fostered compensation mechanisms involving manipulativeness and irresponsibility. Finding solace in experiencing as well as portraying herself as eccentric, M uses alcohol and cannabis as escaping as well as hedonistic behavior. With increasing feelings of staying unduly bereft of life's opportunities as a long term consequence of her strategy, M experiences re-occurring outbursts of anger when things don't go her way on concrete occasions. From this hypothetical case formulation, targeting M's acute moments of emotional turmoil will imply facing up to her hedonistic lifestyle as her long-term dysfunctional coping strategy toward her equally unmet emotional needs.

It is important to note that neither of the presented cases received a PD diagnosis using the categorical diagnostic approach. We used the PID5BF+ M trait model to delineate clinically important personality traits so as to advance our understanding of the how and the why of presenting symptoms, not to unjustly pathologies symptoms that would be deemed subclinical under the DSM-5 Section II classification. For example, L's case formulation allows for an integrated understanding and therapeutic approach that classifying her as having a mood disorder with main concerns related to perfectionism cannot provide for. With the PID5BF+ M as an integration of the DSM-5/ICD-11 trait model, the formal diagnosis of a discrete personality disorder is still dependent upon the patients AMPD Criterion A/ICD-11 Personality Disorder Severity score.

The following limitations need to be considered. First, although the PID5BF+ M represents a solid possible operationalization of the combined ICD-11/DSM-5 Section III model view, the uni- vs. bipolar nature of dimensional scales remains subject of debate (19). Consider for example L's bottom score on Manipulativeness: does this conceptually represent an adaptive absence of manipulativeness, or the presence of a maladaptive gullibility? And in case of the latter, is the trait facet scale able to psychometrically differentiate between both maladaptive extremes on a measurement level? As a second limitation, clinical case reports by definition lack generalizability. However, if general models of personality disturbance have the ambition to claim authority, it is precisely the field of clinical practice that can ultimately test their performance. As a third limitation, the PID5BF+ M does not account for the ICD-11 borderline pattern qualifier. In line with McCabe and Widiger (20) however, we find that the combined ICD-11/DSM-5 trait model is able to adequately account for M's borderline traits. Overall, the current clinical case report illustrates (1) that maladaptive trait expressions are useful sources of information for case formulation, (2) that the Disinhibition/Anankastia personality domain distinction as advocated in the ICD-11 model has clinical utility, (3) that the Psychoticism trait domain as conceptualized in DSM-5 has clinical utility, and (4) that the use of lower order personality trait facets has clinical value beyond trait domains.

## Data Availability Statement

The original contributions presented in the study are included in the article/supplementary material, further inquiries can be directed to the corresponding author/s.

## Ethics Statement

The studies involving human participants were reviewed and approved by Ethisch Comité UPC KU Leuven. The patients/participants provided their written informed consent to participate in this study. Written informed consent was obtained from the individual(s) for the publication of any potentially identifiable images or data included in this article.

## Author Contributions

TB wrote the manuscript. LC and DS carefully read the text and provided suggestions for improvement. All authors contributed to the article and approved the submitted version.

## Conflict of Interest

The authors declare that the research was conducted in the absence of any commercial or financial relationships that could be construed as a potential conflict of interest. The reviewer AK declared a past co-authorship with two of the authors TB, LC to the handling editor.
